# Transfected HEK293 Cells Expressing Functional Recombinant Intercellular Adhesion Molecule 1 (ICAM-1) – A Receptor Associated with Severe *Plasmodium falciparum* Malaria

**DOI:** 10.1371/journal.pone.0069999

**Published:** 2013-07-25

**Authors:** Anja Bengtsson, Louise Joergensen, Zachary R. Barbati, Alister Craig, Lars Hviid, Anja T. R. Jensen

**Affiliations:** 1 Centre for Medical Parasitology at the Department of International Health, Immunology and Microbiology, Faculty of Health and Medical Sciences, University of Copenhagen and at Department of Infectious Diseases, Copenhagen University Hospital (Rigshospitalet), Copenhagen, Denmark; 2 Liverpool School of Tropical Medicine, Liverpool, United Kingdom; Universidade Federal de Minas Gerais, Brazil

## Abstract

Intercellular adhesion molecule 1 (ICAM-1) is a membrane-bound glycoprotein expressed on endothelial cells and cells of the immune system. Human ICAM-1 mediates adhesion and migration of leucocytes, and is implicated in inflammatory pathologies, autoimmune diseases and in many cancer processes. Additionally, ICAM-1 acts as receptor for pathogens like human rhinovirus and *Plasmodium falciparum* malaria parasites. A group of related *P. falciparum* erythrocyte membrane protein 1 (PfEMP1) domains, the DBLβ, mediates ICAM-1 binding of *P. falciparum*-infected erythrocytes. This ICAM‑1-binding phenotype has been suggested to be involved in the development of cerebral malaria. However, more studies identifying cross-reactive antibody and ICAM-1-binding epitopes and the establishment of a clinical link between DBLβ expression and e.g. cerebral malaria are needed before the DBLβ domains can be put forward as vaccine candidates and go into clinical trials. Such studies require availability of functional recombinant ICAM-1 in large quantities. In this study, we compared recombinant ICAM-1 expressed in HEK293 and COS-7 cells with mouse myeloma NS0 ICAM-1 purchased from a commercial vendor in terms of protein purity, yield, fold, ability to bind DBLβ, and relative cost. We present a HEK293 cell-based, high-yield expression and purification scheme for producing inexpensive, functional ICAM‑1. ICAM-1 expressed in HEK293 is applicable to malaria research and can also be useful in other research fields.

## Introduction

ICAM-1 is a member of the immunoglobulin (Ig) superfamily and is expressed by endothelial cells and leucocytes as a membrane-bound protein containing five extracellular Ig-like domains (D1-D5), a trans-membrane domain, and a cytoplasmic domain. ICAM‑1 mediates adhesion and migration of leukocytes by binding to leukocyte function-associated antigen-1 (LFA‑1) and macrophage antigen-1 (Mac-1) [1,2]. It is implicated in inflammatory pathologies, autoimmune diseases, and many cancer processes [3]. It furthermore acts as a receptor for human rhinovirus causing common cold [4–6] and as a receptor for *P. falciparum*-infected erythrocytes (IEs) binding to endothelial cells [7–20].

*P. falciparum* malaria remains a major health issue causing ~200 million cases of disease and ~700,000 deaths annually, mainly among African children below 5 years-of-age [21]. Parasite virulence is closely related to the expression of PfEMP1 on the surface of IEs mediating their adhesion to host endothelium by binding to different vascular host receptors, including ICAM‑1. IE sequestration leads to inflammation, circulatory obstruction, and organ dysfunction [22]. ICAM-1 expressed on vascular endothelial cells has been suggested as a receptor involved in the development of cerebral malaria, a severe and often fatal complication with IE sequestration in the brain [7,9,11,19].

Several ICAM-1-binding PfEMP1 domains and a full length PfEMP1 molecule have previously been characterized [18,23], and we recently identified a conserved domain cassette (DC) structure (DC4) in some of these [20]. DC4-containing PfEMP1 proteins share a particular ICAM‑1-binding phenotype conferred by the DBLβ3_D4 domain of DC4. DC4 has been linked to the pathogenesis of severe disease [24] and can induce cross-reactive adhesion inhibitory antibodies [20]. However, more studies linking ICAM‑1-adhering IEs to severe disease such as cerebral malaria and identifying ICAM-1-binding PfEMP1 epitopes (not least epitopes inducing adhesion-inhibitory antibodies) are needed before DBLβ3_D4 can be put forward as a vaccine candidate. Achievement of this goal depends heavily on the availability of large quantities of high-quality recombinant ICAM-1.

ICAM-1 expressed as a recombinant protein by mouse myeloma NS0 cells can be purchased commercially and has been used in various studies to demonstrate binding of *P. falciparum* IEs to ICAM-1 [13–16]. Other studies have used transfected CHO cells [17,18,20,25]. Finally, COS‑7 cells transiently producing soluble ICAM-1 have also been widely used [8,10,12,26]. Surprisingly, soluble recombinant ICAM-1 expressed in one of the most widely used transient expression systems, human embryonic kidney (HEK) cells and derivatives hereof [27] has only been used for malaria binding assays in very few studies [20,23].

Recombinant protein yield is generally higher in HEK than CHO cells [28], and can reach several hundred milligrams of recombinant protein per litre of culture medium [29,30]. Thus the HEK expression system has the potential to produce large quantities of recombinant ICAM-1 as well as the ability to produce recombinant proteins with appropriate human post-translational modifications.

In this study, we compared ICAM-1 expression in HEK293, COS-7, and mouse myeloma NS0 cells, in terms of protein purity, yield, folding, the ability to bind a recombinant DC4-containing PfEMP1 protein, and relative cost. We present a HEK293 cell-based, high-yield expression and purification scheme for producing inexpensive, functionally intact ICAM-1 able to bind the *P. falciparum* antigen PFD1235w-DBLβ3_D4. 

## Materials and Methods

### Protein expression and purification

Recombinant ICAM-1-Fc chimera (ICAM-1-Fc_HEK293_) was made from expression in FreeStyle 293-F cells (Invitrogen). ICAM-1 D1-D5 combined with the hinge region, CH2 and CH 3 of human IgG1 was cloned into a mammalian expression vector holding a CMV promoter [8]. The vector was amplified in MC1061/P3 *E. coli* cells and DNA was purified using EndoFree Plasmid Maxi Kit (Qiagen). HEK293 cells in the exponential growth phase were grown in Gibco FreeStyle 293 Expression Medium (Invitrogen) until they reached a cell density of 1×10^6^ cells/ml. The cells were transiently transfected using FreeStyle MAX Reagent (Invitrogen) according to the manufacturer’s instructions. Briefly, 120 µg DNA diluted in Gibco OptiPro SFM (Invitrogen) were gently mixed with 120 µl FreeStyle MAX Reagent diluted in OptiPro SFM and incubated for 10 min. The mixture was added drop-wise to a flask containing 150 ml HEK293 cells. The transfected cells were allowed to grow in suspension for six days at 37° C in a humidified atmosphere of 5% CO_2_ on an orbital shaker platform rotating at 135 rpm. Six days following transfection, the HEK293 cells were separated from the ICAM-1-Fc_HEK293_-containing supernatant by centrifugation (20 min, 500 g). The supernatant was filtered (0.2 µm), concentrated, and buffer-exchanged into 20 mM sodium phosphate, pH 7. ICAM‑1-Fc_HEK293_ was bound to a 1 ml HiTrap Protein G HP column (GE Healthcare) connected to an ÄKTAxpress controlled by UNICORN software (GE Healthcare). ICAM-1-Fc_HEK293_ was eluted from the column using Glycine/HCl buffer (0.2M, pH 2.5) and neutralized immediately using Tris/HCl buffer (1M, pH 9.0). Finally, purified ICAM-1-Fc_HEK293_ was buffer-exchanged into PBS. ICAM-1-Fc was also expressed in COS-7 cells (ICAM-1-FC_COS-7_), and purified as previously described [8], where confluent COS-7 cells were transfected using FuGENE6 transfection reagent (Roche) according to the manufacturer’s instructions. Finally, commercially available recombinant ICAM1-Fc (ICAM-1-Fc_NS0_) produced in mouse myeloma cell line NS0 was purchased from R&D Systems.

The ICAM-1-binding DBLβ3_D4 domain and the non-ICAM‑1-binding DBLβ3_D5 domain of the PFD1235w *var* gene from the 3D7 *P. falciparum* parasite [20] were cloned into pET15b modified to encode an N-terminal 6xHis fusion tag, expressed in Shuffle® T7 Express Competent *E. coli* (Medinova), and purified as described [31].

### Sodium dodecyl sulfate polyacrylamide gel electrophoresis (SDS-PAGE)

Recombinant ICAM-1-Fc proteins were separated by SDS-PAGE under reducing (+DTT) and non-reducing conditions (-DTT). Protein (5 µg) was loaded into each well on a NuPAGE® Novex 4-12% Bis-Tris gel in MOPS SDS Running buffer (Invitrogen) and detected using BioSafe Coomassie stain (BIO-RAD). ProSieve Color Protein marker (Lonza) was used for size estimation.

### Dot blot

Dot blots were done by adding a 2-µl drop of purified protein or cell pellet suspended in PBS +2% SDS onto a Hybond^TM^-C Extra membrane (Amersham BioSciences). The membrane was blocked using TBST + 5% skimmed milk (1 h; room temperature). Protein was detected by anti-human IgG-HRP (Dako, 1:3000 in TBST; 1 h; room temperature) using a chemiluminescence detection kit (Pierce).

### Reactivity of monoclonal ICAM-1 *antibodies*

The reactivity of a panel of mouse anti-human ICAM-1 monoclonal antibodies (mAbs) with the ICAM-1-Fc proteins was examined by ELISA. Maxisorp plates (Thermo Scientific) were coated (overnight; 4° C) with ICAM-1-Fc (2 µg/well) in Glycine/HCl buffer (0.1 M, pH 2.75) and blocked with blocking buffer (PBS, 0.5 M NaCl, 1% Triton-X-100, 1% BSA, pH 7.2) for 1 h at room temperature. The following mAbs were added (1 h; room temperature) to each well: RR1/1 (1 µg; AH Diagnostics), 84H10 (0.5 µg; AH Diagnostics), LB2 (0.3 µg; AH Diagnostics), BBIG-I1 (2.5 µg; R&D Systems), 8.4A6 (0.5 µg; SigmaAldrich), My13 (1 µg; Invitrogen) and 15.2 (0.5 µg; AbD serotec). A mouse anti-human CD36 antibody (FA6.152, Ramcon) was included as negative control (2.5 µg). The plates were washed in PBS+1% Triton-X-100, and bound mAbs were detected by anti-mouse Ig-HRP (Dako, 1:1000 in blocking buffer; 1 h). The plates were developed using OPD tablets (Dako) according to the manufacturer’s instructions. The optical density (OD) value was read at 490 nm using a VERSAmax microplate reader (Molecular Devises) and Softmax Pro v 4.7.1.

#### Malaria antigen-binding assay

The malaria antigen binding assay was performed using ELISA as described [20]. Briefly, Maxisorp plates were coated (overnight; 4° C) with recombinant PFD1235w-DBLβ_D4 or DBLβ3_D5 (0.1 µg/well) in Glycine/HCl buffer (0.1 M, pH 2.75) and blocked with blocking buffer (PBS, 0.5 M NaCl, 1% Triton-X-100, 1% BSA, pH 7.2). ICAM-1-Fc (0-1.5 µg/well) was added (1 h; room temperature), followed by washing using PBS+1% Triton-X-100. Bound ICAM-1-Fc was detected by HRP-conjugated anti-human-IgG (1:3000 in blocking buffer; 1 h), and the reaction was developed using OPD as described above. 

## Results and Discussion

### Purity and yield of ICAM-1-Fc

**Figure 1:**
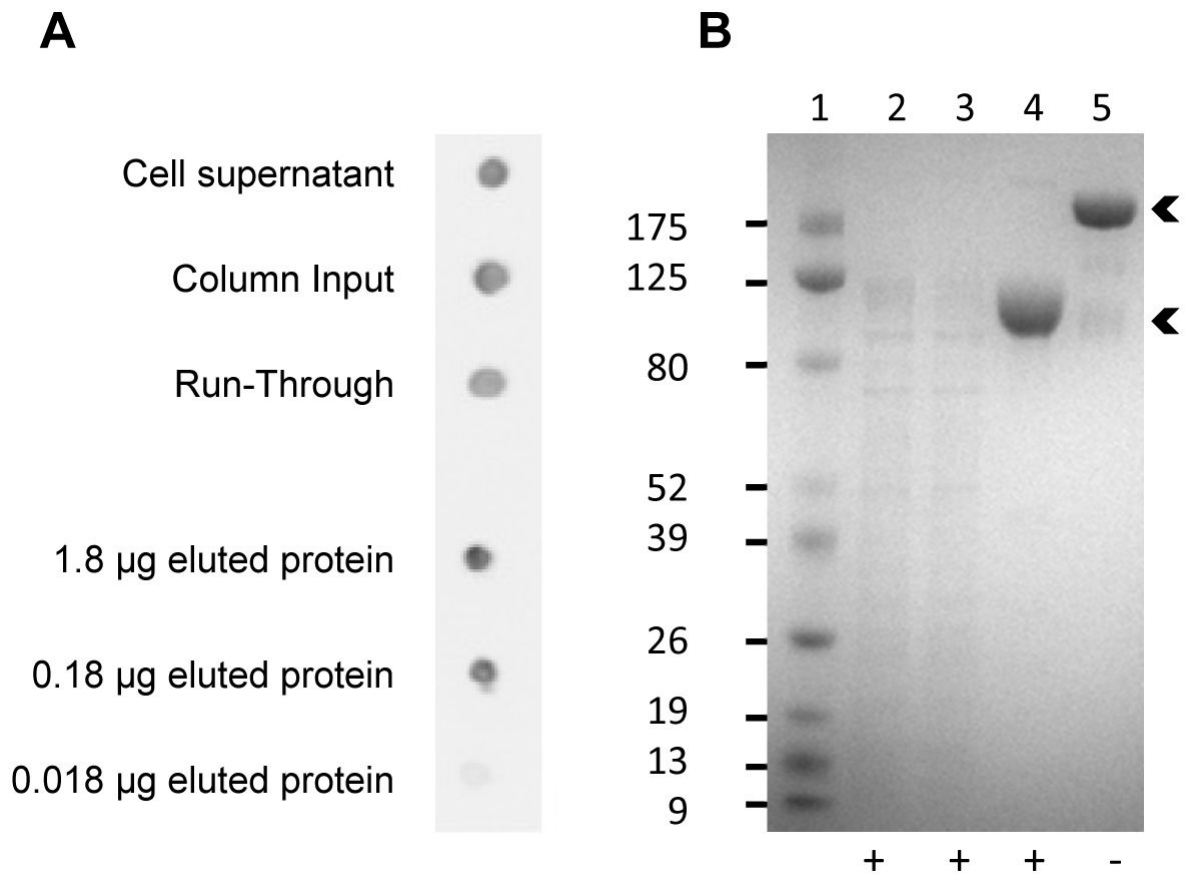
Purification of ICAM1-Fc expressed by HEK293 cells. Data from exp. #1 is shown here as an example of purification of ICAM-1-Fc_HEK293_. (**A**) Dot blot showing 2 µl of cell supernatant at day of harvest, 2 µl diafiltrated supernatant (column input) and 2 µl column run-through. 1.8 µg and two ten-fold dilutions hereof of the eluted ICAM-1-Fc was dotted onto the membrane. ICAM-1-Fc was detected using HRP-conjugated anti-human IgG antibody. (**B**) Sodium dodecyl sulfate polyacrylamide gel electrophoresis (SDS-PAGE) gel electrophoresis of 5 µl protein marker (lane 1), 10 µl column input (lane 2), 10 µl column run-through (lane 3) and 9 µg eluted ICAM-1-Fc (lane 4+5). Samples were reduced using DTT (+) or non-reduced (−). Arrows indicate ICAM-1-Fc bands.

Transfected HEK293 cells were grown for 6 days before harvesting ICAM-1-Fc_HEK293_ from the supernatant. Supernatant ICAM-1-Fc_HEK293_ could be detected by dot blotting before and after buffer exchange and concentration (Figure 1A) but was not visible by SDS-PAGE (Figure 1B, lane 2). Despite the presence of protein contaminants in the supernatant before purification, ICAM-1-Fc_HEK293_ was the major protein species eluted from the Protein G column yielding very pure protein (Figure 1B, lanes 4–5). Purified ICAM-1-Fc_HEK293_ was separated both under reducing and non-reducing conditions on the SDS-PAGE gel. A band shift was observed from ~100 kDa under reducing to ~200 kDa under non-reducing conditions (Figure 1B, lanes 4-5). The protein size under reducing conditions was larger than the estimated molecular weight (79 kDa) of ICAM-1-Fc estimated from the protein sequence (http://web.expasy.org/protparam/). Each ICAM-1 molecule has eight potential N-glycosylation sites of which seven of them are glycosylated when expressed in CHO cells [32]. The increased molecular weight observed here is thus most probably due to glycosylation of these sites. The difference between theoretical and observed molecular weight corresponds well to the sizes of the commercially available ICAM-1_NS0_ given by the manufacturer. Under non-reducing conditions, the ICAM-1-Fc is expected to be a dimer due to a disulfide bond between the two Fc domains. This prediction corresponds well with the observed molecular weight (~200 kDa) for the non-reduced ICAM-1-Fc.

ICAM-1-Fc_HEK293_ was expressed twice with cells grown in four flasks containing 150 ml of culture. Cells from the four flasks were pooled, centrifuged, and discarded. The supernatant was buffer-exchanged and reduced to a volume of 200 ml, which was loaded onto the protein G column. The yields of ICAM-1-Fc_HEK293_ in the two experiments were 7.1 mg and 7.0 mg, respectively, corresponding to a yield of 11.8 mg/L and 11.6 mg/L. Re-purification on a protein G column of the ICAM-1-Fc_HEK293_ remaining in the column run-through (Figure 1A) yielded an additional 4.0 mg protein. Thus, the averaged total protein yield was 15.1 mg/L. Subsequent expression runs gave similar protein yields (data not shown). In comparison, expression in COS-7 cells gave 1.3 mg ICAM-1-Fc per litre cell culture supernatant (data not shown). The expression and purification data of soluble ICAM1 in COS-7 cells have been previously published [8].

### Folding of ICAM-1-Fc_HEK293_ resembles reference ICAM-1-Fc

**Figure 2:**
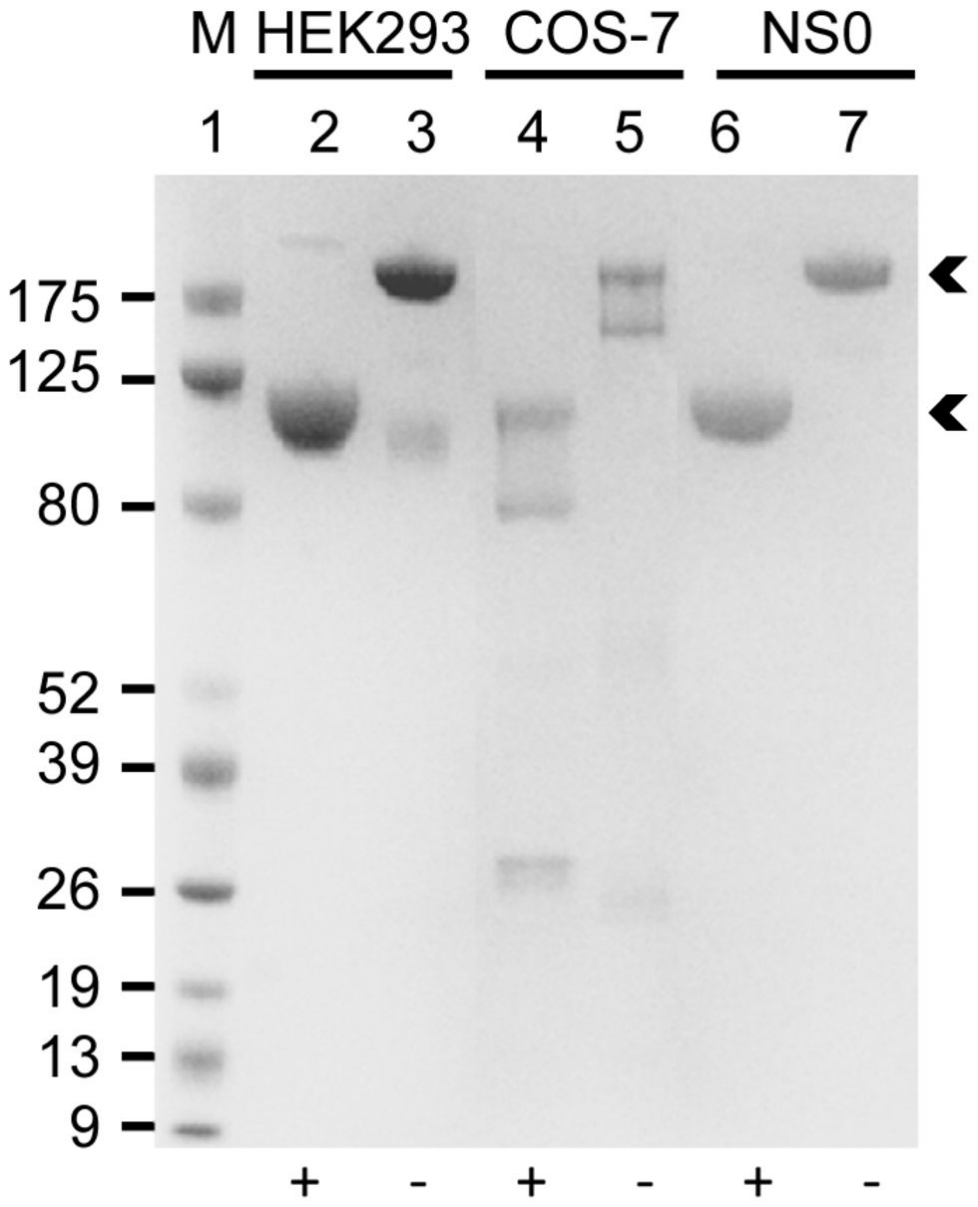
Comparison of ICAM-1-Fc by Sodium dodecyl sulfate polyacrylamide gel electrophoresis (SDS-PAGE). SDS-PAGE gel electrophoresis of 5 µg of ICAM-1-Fc expressed in HEK293 cells, COS-7 cells or in mouse myeloma NS0 (R&D Systems) cells. 5 µl protein marker (M) was loaded onto the gel. Samples were reduced using DTT (+) or non-reduced (−). Arrows indicate ICAM-1-Fc bands.

**Figure 3:**
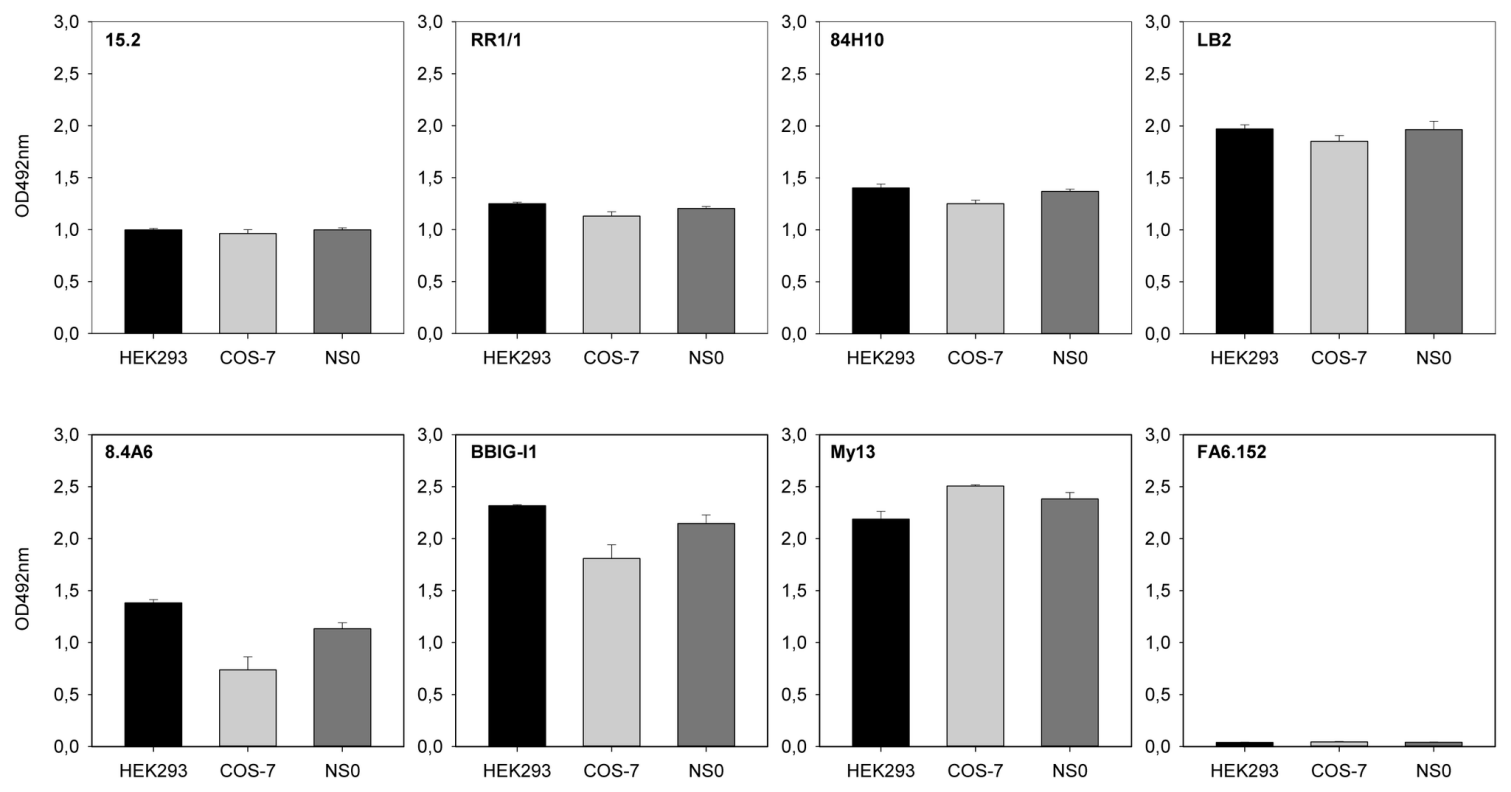
Reactivity of monoclonal ICAM antibodies. The reactivity of seven anti-human ICAM-1 specific monoclonal antibodies (clones 15.2, RR1/1, 84H10, LB2, BBIG-I1, 8.4A6 and My13) against ICAM-1-Fc expressed in HEK293, COS-7 or mouse myeloma NS0 (R&D Systems) cells were tested using ELISA. One CD36 specific monoclonal antibody (clone FA6.152) was included as negative control. Data shown are the mean reactivity (three independent experiments) of the antibodies to ICAM-1. Errors indicate S.D.

ICAM-1-Fc expressed in COS-7 cells and in mouse myeloma NS0 cells have previously been shown to function as receptors for PfEMP1 [8,10,12–16,26]. We therefore compared our ICAM-1-Fc_HEK293_ and ICAM-1-Fc_COS-7_ to commercial ICAM-1-Fc_NS0_ by SDS-PAGE and by their ELISA reactivity with a panel of seven human ICAM-1-specific mouse mAbs. All three proteins behaved similarly on SDS-PAGE showing the expected molecular sizes under reducing (monomer) and non-reducing (dimer) conditions (Figure 2). Contaminating bovine IgG from the culture medium was present in ICAM-1-Fc_COS-7_ (Figure 2, lanes 4-5) and thus contributes to the 5 µg of total protein loaded onto the gel. The proteins were recognized equally well by all seven ICAM-1-specific mAbs, except for 8.4A6, which reacted less well with ICAM-1-Fc_COS-7_ (Figure 3). The mAbs 15.2, RR1/1, 84H10, LB2, BBIG/I1 and My13 recognize conformational epitopes in the first Ig domain of ICAM-1 [26,33,34], whereas mAb 8.4A6 recognizes a linear penta-peptide present in the second Ig domain of ICAM-1 [34]. We conclude that the conformation of ICAM-1-Fc_HEK293_ corresponds to that of ICAM-1-Fc_COS‑7_ and ICAM-1-Fc_NS0_.

### HEK293 cells produce functionally intact ICAM-1-Fc binding a malaria antigen

**Figure 4:**
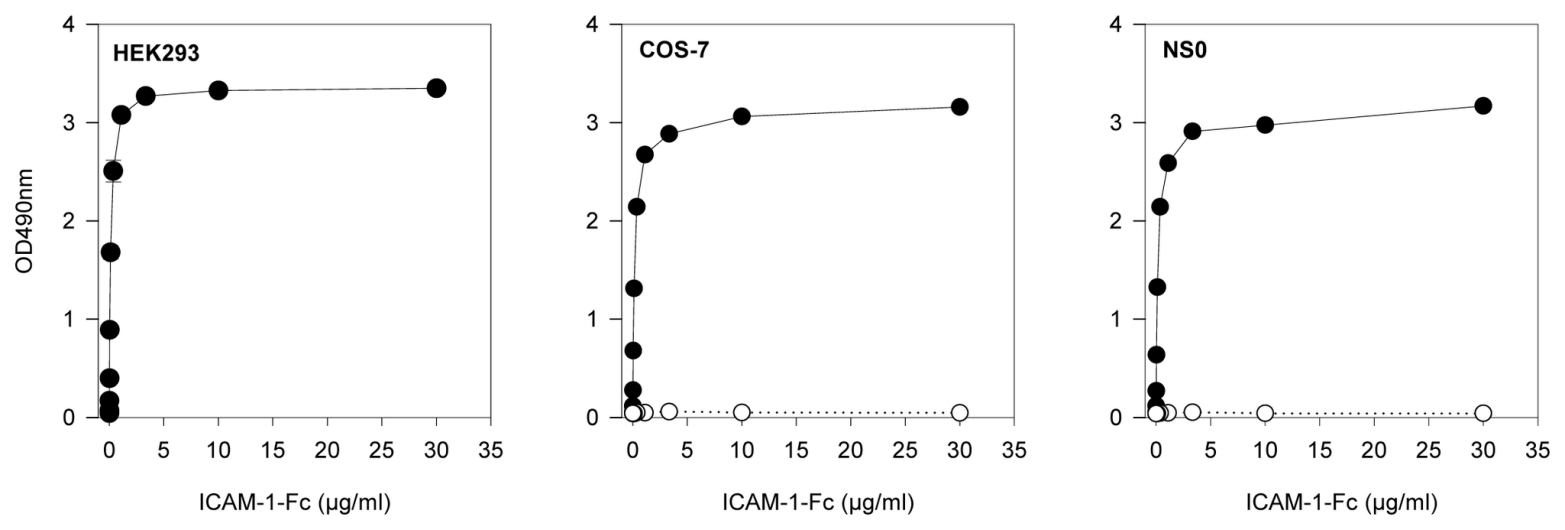
Binding of the malaria PfEMP1 antigen DBLβ3_D4 to ICAM-1-Fc. Concentration-dependent binding of recombinant *P. falciparum* 3D7 PFD1235w DBLβ3_D4 to ICAM-1-Fc_HEK239,_ ICAM-1-Fc_COS-7_ and ICAM-1-Fc_NS0_ (R&D Systems) by ELISA. The binding of DBLβ3_D4 to ICAM-1-Fc_HEK239_ was repeated in three independent experiments (mean and standard deviation shown) while the assay using ICAM-1-Fc_COS-7_ and ICAM-1-Fc_NS0_ (R&D Systems) was done one time each.

Binding parameters of a single PfEMP1 domain has previously been shown to be similar to that of a IT4 PfEMP1 ectodomain [23]. We have previously shown that the DBLβ3_D4 domain of the PfEMP1 protein PFD1235w binds to ICAM‑1, whereas the immediately downstream DBLβ3_D5 domain does not [20]. ICAM-1-Fc_HEK293_ expressed in this study was fully functional and bound to DBLβ3_D4 in a concentration-dependent manner (Figure 4). In contrast, DBLβ3_D5 did not bind to any of the ICAM‑1 constructs (Figure 4). The molecular weights of all the ICAM-1 constructs were the same, but ~20 kDa bigger than predicted, probably due to glycosylation of the N-glycosylation sites. The nature of sugar chains added to ICAM-1 differs significantly between glycosylation site and the ICAM-1 expressing cell, hence between expression systems [28,32]. These differences affect the binding of ICAM-1 to some receptors (e.g., Mac-1) but not others (e.g., LFA‑1) [35] and might regulate the biological activity of ICAM-1 *in vivo* [32]. However, the role of ICAM-1 glycosylation in *P. falciparum* infections remains to be investigated. The glycan profile of ICAM-1_HEK239_ has been shown similar to that of ICAM-1_COS-7_ and less variant than that of ICAM-1_NS0_ [29], but from our experiments these differences did not seem to affect the ICAM-1 binding to DBLβ3_D4.

### Expression of ICAM-1-Fc in HEK293 cells is cost-effective

As reported above, we achieved yields of ICAM-1-Fc_HEK239_ that were 10-fold higher than yields of ICAM-1-Fc_COS-7_. This makes the HEK239 system very cost-effective. In our hands, the cost of producing ICAM-1-Fc_HEK239_ is about 24-fold lower than ICAM-1-Fc_COS-7,_ and about 90-fold lower than buying ICAM-1-Fc from a commercial source (e.g. R&D Systems, list price DKK. 2320). This estimate is based on the cost of cell lines, culture flasks, media, transfection reagents, and columns for purification, salaries and the needed equipment. It furthermore assumes that standard laboratory equipment such as laminar flow hoods, CO_2_ incubators, orbital shakers, and a system for protein purification are available.

## Conclusion

In this study we present a high-yield expression and purification scheme for producing inexpensive, functionally intact ICAM-1-Fc in transfected HEK293 cells. In addition to being useful in malaria research, the HEK239 cell-produced protein might also be useful in other research areas, as ICAM‑1 also acts as a receptor for cells of the immune system and viruses such as human rhinovirus.
